# A Review Leveraging a Rare and Unusual Case of Basal Cell Carcinoma of the Prostate

**DOI:** 10.1155/2021/5520581

**Published:** 2021-05-04

**Authors:** Lin He, Christopher Metter, Vitaly Margulis, Payal Kapur

**Affiliations:** ^1^Department of Pathology, University of Texas Southwestern Medical Center, Dallas, TX 75390, USA; ^2^Department of Urology, University of Texas Southwestern Medical Center, Dallas, TX 75390, USA

## Abstract

Basal cell carcinoma (BCC) is a rare nonacinar variant of prostatic carcinoma. In spite of prostatic acinar adenocarcinoma being one of the most common carcinomas in prostate, <100 prostatic BCC cases have been reported to date. Adenoid cystic/cribriform histology has been described in varying proportions to occur in prostatic BCC and is reported to be associated with aggressive behavior and high risk of metastasis. Herein, we present a case of prostatic BCC with adenoid cystic morphology, comprehensively describe its immunohistochemical and *MYB/MYBL1* gene rearrangement findings, discuss its differential diagnosis, and review the literature of this rare entity.

## 1. Introduction

Basal cell carcinoma (BCC) is a rare malignant neoplasm of the prostate that is composed of prostate basal cells [1]. The first case of prostatic BCC was published in 1974 [2] and less than 100 BCC of prostate have been described to date [3–13]. Morphologically, it shares features to the cutaneous basal cell carcinoma and includes nests of basaloid cells with hyperchromatic nuclei and scant cytoplasm, tumor necrosis, and peripheral palisading. Prostatic BCC was initially referred to as adenoid cyst carcinoma (ACC) of the prostate due to the presence of cribriform pattern with intraluminal eosinophilic hyalinized substance and its resemblance to the salivary gland and breast counterparts [1]. In 2004, the World Health Organization (WHO) recognized ACC of the prostate as a spectrum of prostatic BCC [14]. Prostatic BCC without cribriform morphology can have overlapping features to a spectrum of benign entities in the prostate, such as florid basal cell hyperplasia [15]. For such morphologically challenging cases, immunohistochemical (IHC) stains including Bcl-2 and Ki-67 can aid in the diagnosis [16]. However, due to its rarity, genetic and molecular characteristics of prostatic BCC have not been extensively studied. Similar to the ACC of the salivary gland, recurrent *t* (6; 9) translocation, resulting in a fusion of *MYB* oncogene to the transcription factor gene *NFIB*, has been observed in a subset of prostatic BCC with ACC morphology [4]. Herein, we presented an unusual case of prostatic BCC with mixed ACC and non-ACC histologies and present its immunohistochemical profile and *MYB* rearrangement findings.

## 2. Case Presentation

A 92-year-old male with a past medical history of chronic heart failure and coronary artery disease presented with urethral stricture, concerning for urinary retention. He had a long urological history of membranous urethral stricture, and urinary retention which was managed by urethral dilations and green light laser prostatectomy since 2018. Cystoscopy was attempted but failed due to urethral stricture. CT imaging of the pelvic area revealed diffuse heterogeneous appearance of the prostate gland with suspicion for involvement of the bladder neck. The patient underwent transurethral resection of prostate (TURP) for both diagnostic and therapeutic purposes, and TURP specimen was sent to pathology. Post-TURP serum prostate-specific antigen (PSA) was within the normal range (<0.05 ng/mL).

**Figure 1:**
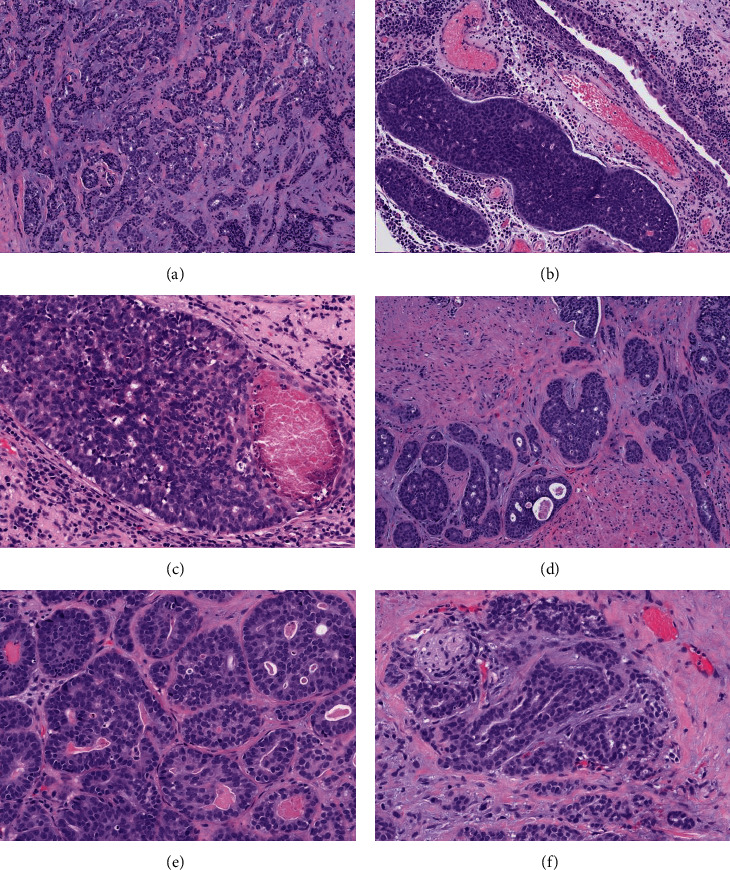
Representative hematoxylin and eosin images (a–f) revealing tumor composed of variably sized irregular solid nests of basaloid cells and anastomosing tubules with desmoplastic stroma (a, 100x magnification). Focal peripheral palisading with peritumoral clefting (b, 100x magnification), comedonecrosis (c, 200x magnification), and invasion into the bladder neck (d, 100x magnification) was noted. Cribriform architecture with eosinophilic hyaline material in the lumen resembling adenoid cystic carcinoma (e, 200x magnification) and perineural invasion (f, 200x magnification) were also present.

On gross examination, the specimen consisted of multiple tan-pink rubbery cauterized tissue fragments, weighing 3 grams and measuring 5.8 × 2.3 × 0.2 cm in aggregate. Microscopically, the prostate chips showed variable sized medium to large irregular shaped basaloid nests (Figure 1(a)). Focal areas with peripheral palisading (Figure 1(b)), anastomosing nests (Figure 1(a)), and tubules with necrosis were seen (Figure 1(c)). Some of the nests were present in between benign prostate glands and focally extended between thick muscle bundles suggestive of invasion into the bladder neck (Figure 1(d)). Adenoid cystic-like pattern with cribriform architecture, intra-luminal eosinophilic, hyaline basement membrane-like material (Figure 1(e)), and perineural invasion (Figure 1(f)) was also present. The tumor cells had scant cytoplasm, irregular angulated nuclei with open chromatin, and high nuclear to cytoplasmic ratio. Focal stromal desmoplastic response was observed (Figure 1(a)). The tumor extensively involved 30% of the chips and focally spread to the overlying urothelium.

**Figure 2:**
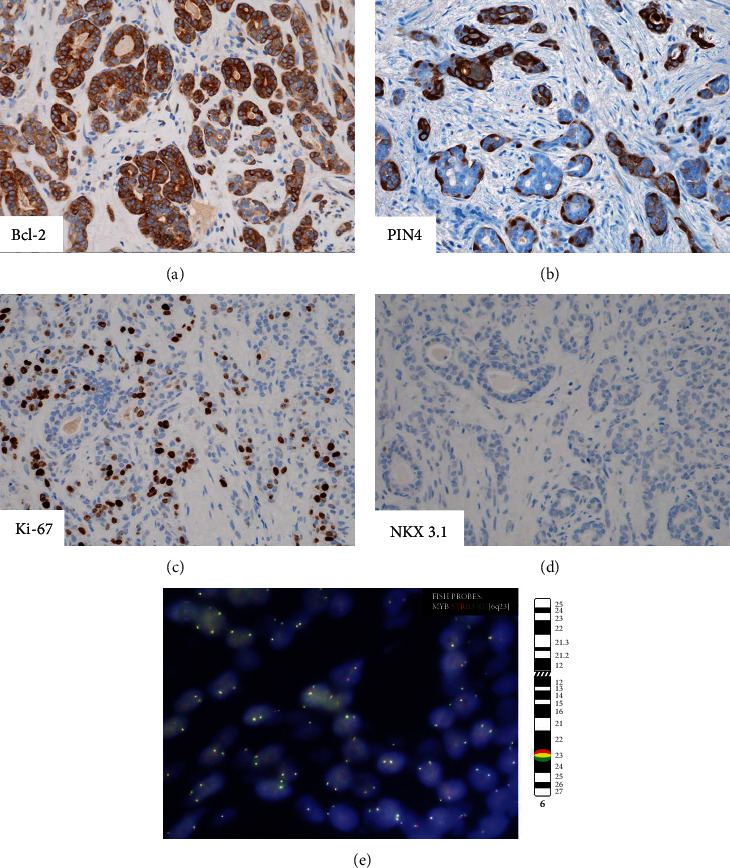
Immunohistochemistry and cytogenetics studies support the diagnosis of prostatic BCC. The tumor cells revealed strong and diffuse reactivity for Bcl-2 (a, 200x magnification), variable multilayer reactivity for basal markers such as p63 and HMWCK (b, 200x magnification). Ki-67 staining showed high proliferation of >30% (c, 200x magnification). The tumor was negative for NKX3.1 (d, 200x magnification) and racemase (b, 200x magnification). Break-apart FISH for *MYB* image did not show separation of *5*′*MYB* and *3*′*MYB* (E).

Immunohistochemically, the tumor revealed strong and diffuse reactivity for cytokeratin (CK) 7 and Bcl-2 (Figure 2(a)) and weak focal staining for GATA3. Variable multilayer reactivity for basal markers such as p63 and high molecular weight cytokeratin (HMWCK) was observed (Figure 2(b)). Ki-67 staining showed high proliferation (>30%, Figure 2(c)). The tumor was negative for CK20, NKX3.1 (Figure 2(d)), racemase (Figure 2(b)), synaptophysin, chromogranin, androgen receptor (AR), and HER2 (Table 1). These histopathologic and IHC features are compatible with the diagnosis of prostatic BCC.

*MYB* gene rearrangement by fluorescence in situ hybridization (FISH) using a break-apart probe was performed. In spite of a prominent ACC-like pattern, *MYB* break-apart was negative/not detected and all tumor cells have two fusion fluorescence signals (Figure 2(e)). Additional FISH studies using two fusion probes for MYB-NFIB and MYBL1-NFIB fusions also turned out to be negative.

Due to the patient's age and systemic conditions, resection surgery was not deemed ideal, and the patient was treated with external beam radiotherapy to prevent morbidity from local disease progression and potentially to further relieve his urinary obstruction symptoms. The patient is currently alive 4 months post-diagnosis.

## 3. Discussion

We report a rare case of prostatic BCC and discuss the differential diagnosis and immunohistochemical profile that can help establish the correct diagnosis.

Prostatic BCC is composed of neoplastic prostatic basal cells unlike the more common variant of prostatic acinar adenocarcinoma that arises from the secretory epithelial cells of the prostate ducts and acini. The number of basal cells is significantly less compared to secretory cells in the prostate gland, perhaps explaining why prostatic BCC is rare with approximately 100 cases reported so far. Similar to our case, they have largely been reported in older men (42-93 years) [3, 8, 10, 17] with urinary obstruction symptoms. Since it is the secretory cells that contain and secrete prostate-specific antigen (PSA), the serum PSA levels in these patients are generally within normal range [3, 8, 18] unless there is concomitant acinar carcinoma which may be found in a minority of cases [3, 8, 10, 19]. The symptoms are therefore often misinterpreted to be due to benign prostate hyperplasia, and in the majority of the cases, the diagnosis is made on transurethral resection specimen [8, 9].

Similar to our cases, most cases can show different histologies. The two common patterns that have been reported in literature include the following: (1) adenoid cystic/cribriform pattern with luminal inspissated secretions and (2) basaloid pattern with small nests of basal cells. Infrequently, small tubules lined by a hyaline rim and cords of tumor cells can also be seen. These patterns can occur either exclusively or in varying proportions in an individual case. Tumors with predominant adenoid cystic pattern can be more easily recognized as malignant and resemble ACC of the salivary glands and the breast [2, 6, 12, 13, 20, 21]. BCC can mimic florid basal cell hyperplasia and can be diagnosed on the bases of cytoarchitectural atypia, invasive pattern into stroma and between normal prostate acini, perineural invasion, luminal necrosis, and extraprostatic extension [6, 7, 22].

Immunohistochemistry can aid in the differential diagnosis that includes, florid basal cell hyperplasia, high-grade prostatic acinar adenocarcinoma, neuroendocrine carcinoma, urothelial carcinoma, and metastasis. Basal cell markers such as HMWCK (34*β*E12) and p63 [23] often label the outermost layers of BCC and may not stain the luminal cells (that may stain with cytokeratin 7) [8] (Table 2). It has been shown that in 60% (15/25) cases, multiple layers of cells except the innermost luminal layer express basal markers, and in 24% (6/25), only the outermost layer expresses basal cell markers, while in 16% (4/25), only a few scattered positive cells are identified [3]. This could lead to misinterpretation in the diagnosis. The strong Bcl-2 expression and increased Ki-67 (often >20%) can help in distinguishing it from florid basal cell hyperplasia [16]. Negative staining for markers expressed in secretory cells such as PSA, prostatic specific acid phosphatase (PSAP), NKX3.1, AMACR, androgen receptor (AR) [3, 17, 19], and ERG [19] can help differentiate it from prostatic acinar adenocarcinoma. Negative GATA3 can help exclude urothelial carcinoma and prostate adenocarcinoma [24]. Weak GATA3 staining has been reported in occasional benign prostatic basal cells [25]. In our case, the tumor cells also show weak positivity for GATA3. We are not aware of other studies that show a similar weak positivity pattern of GATA3 or lack of NKX3.1 in prostatic BCC cases. Negative neuroendocrine markers help distinguish it from neuroendocrine carcinoma. HER2 expression may be variable in prostatic BCC and may provide a therapeutic opportunity [22, 26]. However, HER2 was negative in our case. Table 2 summarizes studies of the prostatic BCC and their morphologic and IHC results [2–13, 17, 20, 21, 27–45].

*MYB-NFIB* fusion has been recognized as a hallmark genetic driver for ACCs of various anatomic sites including salivary gland [46, 47], breast [48, 49], lacrimal gland [50], and skin [51]. Akin to ACC in the salivary glands, 17-47% of prostatic BCC have been shown to harbor the *MYB-NFIB* fusion [4, 9, 19, 52] by FISH techniques. The majority (57%) of these cases had ACC morphology. In contrast, only 93% of fusion negative cases had ACC features. *MYB* rearrangement using the break-apart probe was not detected in our case in spite of the presence of ACC morphology. The detection of the fusion may vary depending on the probe used. Using a break-apart probe, Bishop et al. detected *MYB* rearrangement in 29% (2/7) [4], while Magers et al. found fusions in 89% (8/9) using an *MYB-NFIB* fusion probe [9]. *MYBL1* variant has been reported to be an alternative genetic driver for ACC in the breast [53]. This discrepancy may also be the result of a small sample size. None of the cases with benign basal cell proliferations were found to have this fusion [9]. *TMPRSS2-ERG* fusions, which are seen in 50% of the prostatic adenocarcinoma, have not been reported in BCC [19]. In our case, neither MYB rearrangement nor MYB/MYBL1-NFIB fusion is present.

BCC of the prostate is reported to be more aggressive than acinar adenocarcinomas. Extraprostatic extension at radical prostatectomy is reported in 44-71% [3, 9], distant metastasis in 14-29%, and disease associated died in 50% [3, 8, 10] of patients. The metastasis pattern is also different from the traditional prostatic adenocarcinoma. Prostatic BCC tends to metastasize to the liver and lung, though bone metastasis has also been reported [5, 8]. Ablative therapy is recommended [5, 8] for these patients.

In summary, prostatic BCC can be challenging to diagnose. Better awareness of this entity, establishing basal cell immunophenotype, and identification of malignant features such as cytologic atypia, unequivocal invasion, perineural invasion, necrosis, or extraprostatic extension can aid in making the correct diagnosis.

## Figures and Tables

**Table 1:** Immunohistochemical antibody information.

Antibody	Source	Dilution	Immunoreactivity
p16	VENTANA	PD∗	Positive
NKX3.1	CELL MARQUE	PD	Negative
CK7	VENTANA	PD	Positive
CK20	VENTANA	PD	Negative
Synaptophysin	VENTANA	PD	Negative
Chromogranin	VENTANA	1 : 2	Negative
HMWCK	VENTANA	PD	Positive
p63	VENTANA	PD	Positive
Racemase	ZETA CORP	1 : 200	Negative
Gata3	CELL MARQUE	PD	Weakly positive
Androgen receptor	VENTANA	PD	Negative
HER2	VENTANA	PD	Negative
Bcl-2	VENTANA	PD	Positive
Ki-67	VENTANA	PD	>30%

^∗^PD: prediluted (ready to use).

**Table 2:** A summary of prostatic BCC studies (empty cells: no information was available).

Year	Study	Sample size	Morphology			IHC										FISH	
			ACC-like pattern	Desmo-plasia	PNI	CK7 (+)	CK20 (+)	HMWCK (+)	p63 (+)	Race-mase (+)	NKX3.1 (+)	PSA (+)	PSAP (+)	Bcl-2 (+)	Ki-67 >20%	MYB rearrangement	MYB-NFIB fusion
Case series with>1 case report^∗^																	
1975	Tannerbaum [12]	2	100%														
1984	Reed [11]	4	100%														
1988	Young [13]	2	100%									50%	50%				
1988	Grignon [7]	2	100%		50%			100%				0%	0%				
1993	Devaraj [6]	4	100%		25%			100%				50%	50%				
2003	Iczkowski [8]	19	63%		26%	100%	0%	100%	86%	0%		20%	13%				
2004	McKenny [10]	4	75%	0%	100%			75%	75%			0%					
2007	Ali [3]	29	64%	71%	14%				100%	27%				92%	57%		
2013	Chang [5]	3	0%					100%	100%					100%	100%		
2015	Bishop [4]	12	58%													17%	
2019	Magers [9]	30	64%	14%	64%												47%
Single case report																	
1974	Frankel [2]	1	+														
1978	Kramer [35]	1	+		+												
1984	Kuhajda [36]	1	+		–							–	–				
1984	Chan [29]	1	+		–												
1986	Gilmour [31]	1	+														
1991	Ahn [27]	1	+		+												
1992	Denholm [21]	1	–	–	–			+				–	–				
1993	Cohen [20]	1	+									–	–				
1996	Hasan [32]	1	–														
2001	Minei [38]	1	+									–	–				
2002	Schmid [41]	1	+		+												
2003	Mastropasqua [37]	1	–		+	–	–	+	+			–	–				
2004	Parada [39]	1	–	+				+				–	–				
2004	Tulunay [45]	1	+					+				–	–				
2006	Fayyad [30]	1	+			+	–	+				–		Weak+			
2008	Hudson [33]	1	+					+	+			–		+	+		
2008	Segawa [42]	1	–			–	–	+	+			–	Weak+				
2010	Bohn [28]	1	+		+	+	–	+	+			–	–	Focal+	20%		
2010	Komura [34]	1	–			+	–	+	+			–	Weak+				
2012	Stearns [43]	1	–														
2013	Rodriguez-Carlin [40]	1	+		+			+	+					+			
2014	Tsuruta [44]	1	–				+	+	+					+	10%		
2019	Shibuya [17]	1	–						+				–				
Current case report		1	+	+	+	+	–	+	+	–	–			+	30%	–	–

^∗^Some of these case series may have overlapped cases.
